# Glycemic variability in normal glucose tolerance women with the previous gestational diabetes mellitus

**DOI:** 10.1186/s13098-015-0077-5

**Published:** 2015-09-24

**Authors:** Yong-mei Wang, Li-hua Zhao, Jian-bin Su, Hai-feng Qiao, Xiao-hua Wang, Feng Xu, Tong Chen, Jin-feng Chen, Gang Wu, Xue-qin Wang

**Affiliations:** Department of Gynaecology and obstetrics, The Second Affiliated Hospital of Nantong University, No. 6 North Hai-er-xiang Road, Nantong, 226001 China; Department of Endocrinology, The Second Affiliated Hospital of Nantong University, No. 6 North Hai-er-xiang Road, Nantong, 226001 China; Department of Clinical Laboratory, The Second Affiliated Hospital of Nantong University, No. 6 North Hai-er-xiang Road, Nantong, 226001 China

**Keywords:** Glycemic variability, Normal glucose tolerance, Gestational diabetes mellitus

## Abstract

**Background:**

Women with previous gestational diabetes mellitus (pGDM) and postpartum normal glucose tolerance (NGT) may carry impaired islet *β* cell secretion, insulin resistance and subsequent altered glucose homeostasis. And certain normoglycemic groups at risks of diabetes were presented with elevated glycemic variability. The aim of study was to investigate the glycemic variability in NGT women with pGDM.

**Methods:**

Total 48 NGT women with pGDM (pGDM group) and 48 age- and BMI-matched NGT women without pGDM (control group) were recruited in the study. Integrated *β* cell function was assessed with the Insulin Secretion-Sensitivity Index-2 (ISSI-2) derived from oral glucose tolerance test. All subjects were monitored using the continuous glucose monitoring system for consecutive 72 h. The multiple parameters of glycemic variability included the mean blood glucose (MBG), standard deviation of blood glucose (SDBG), mean of daily differences (MODD), mean amplitude of glycemic excursions (MAGE) and the incremental areas above preprandial glucose values (AUC_pp_).

**Results:**

The pGDM group had a higher MBG (6.5 ± 0.9 vs. 5.9 ± 0.8 mmol/L, *p* < 0.05), SDBG (1.3 ± 0.3 vs. 0.9 ± 0.2 mmol/L, *p* < 0.05), MODD (1.4 ± 0.3 vs. 1.1 ± 0.2 mmol/L, *p* < 0.05), MAGE (2.7 ± 0.4 vs. 1.8 ± 0.5 mmol/L, *p* < 0.05), and AUCpp (26.8 ± 3.4 vs. 19.2 ± 3.2 mmol/L·h, *p* < 0.05), when compared to the control group, and the differences remained significant after adjusting for anthropometric indices and metabolic risk factors. Islet *β* cell function index ISSI-2 in the pGDM group was lower than in the control group (*p* < 0.05). MBG, SDBG, MODD, MAGE and AUC_pp_ were all negatively associated with ISSI-2 in the pGDM group (*r* = −0.31, −0.30, −0.34, −0.48 and −0.54, respectively, *p* < 0.05), and the correlations remained significant after adjusting for anthropometric indices and metabolic risk factors.

**Conclusions:**

Normal glucose tolerance women with pGDM were presented with elevated glycemic variability, which may be associated with impaired islet *β* cell function.

## Background

Gestational diabetes mellitus (GDM) was associated with increased risks of developing postpartum pre-diabetes, type 2 diabetes and metabolic syndrome [1, 2]. The relationships reflected the fact that GDM and type 2 diabetes shared a similar pathophysiology, characterized by deficiency in islet *β* cell secretion and insulin sensitivity [3, 4]. GDM may serve as a window to reveal a predisposition to type 2 diabetes.

It also had been repeatedly demonstrated that women with previous GDM (pGDM) and even with postpartum normal glucose tolerance (NGT) carried deficiency in islet *β* cell secretion and insulin sensitivity and subsequent altered glucose homeostasis [5–7]. Definition of NGT was based on fasting plasma glucose (FPG) <5.6 mmol/L and 2-h postload plasma glucose (2hPG) <7.8 mmol/L during a 75-g oral glucose tolerance test (OGTT) [8]. Detection of glucose values at 0 and 120 min by OGTT may miss the potential glycemic variability. And previous studies conducted in NGT subjects at risks of developing diabetes showed that glycemic excursions were in the range of prediabetes as well as the diabetes [9, 10]. So we hypothesized that women with pGDM and even with NGT status may have altered glycemic variability.

Efforts to quantify glycemic variability mainly relied on intermittent glucose determinations which acquired from the continuous glucose monitoring (CGM) system (CGMS), and CGM system can detect glycemic variability in more details than the conventional self-monitoring methods of blood glucose [11, 12]. Glycemic variability parameters estimated by the multiple modalities of CGM data [13] may differ in NGT women with and without the pGDM.

The present study was designed to investigate the glycemic variability assessed by CGM in postpartum NGT women with the pGDM.

## Methods

### Study subjects

This cross-sectional study was performed at outpatient and inpatient department of the Second Affiliated Hospital of Nantong University in China from January 2010 to May 2014. Total 502 women who had the diagnosis of GDM between 24 and 28th week of pregnancy were revisited and screened by 75-g oral glucose tolerance test (OGTT) after delivery for 1 year. And 48 women, who detected with postpartum NGT and agreed to be performed with CGM, were recruited for the further study (pGDM group). The diagnoses of NGT and GDM were based on the criteria of the ADA 2008 [8]. NGT was defined as FPG <5.6 mmol/L and 2hPG <7.8 mmol/L during the OGTT. GDM was made when two of the following plasma glucose values in the 75-g OGTT are exceeded: fasting, 5.3 mmol/L; 1 h, 10.0 mmol/L; 2 h, 8.6 mmol/L. Meanwhile, 48 healthy women without pGDM, who selected and diagnosed with NGT after delivery for 1 year in the same department, were recruited and set as controls in the study (control group). The two groups were also matched for age and body mass index (BMI). And exclusion criteria for control group were as follows: family history of diabetes, lipid abnormalities, hypertension, hepatic disease, chronic kidney disease, cardiovascular disease, malignancy, or other disorders affecting glucose metabolism such as hyperthyroidism. The study was approved by the institutional review board of the Second Affiliated Hospital of Nantong University, with written informed consent being obtained from all participants.

### *β* cell function determination

Blood samples were taken at 0, 30, 60, 120 and 180 min for the measurement of plasma glucose and insulin concentrations (glucose unit: mmol/L, insulin unit: miu/L) during 75-g oral glucose test. Glu*t* and Ins*t* represent the plasma glucose and insulin concentrations, respectively, at time *t* during the OGTT. Insulin sensitivity was estimated using the insulin sensitivity index (ISI) of Matsuda and DeFronzo: ISI = 10,000/square root of (Ins0 × Glu0) × (mean glucose × mean insulin during OGTT) [14]. Insulin secretion was defined as the ratio of the area-under-the-insulin-curve to the area-under-the-glucose curve (AUC_ins/glu_) [15, 16]. Integrated *β* cell function was assessed with the Insulin Secretion-Sensitivity Index-2 (ISSI-2) (AUC_ins/glu_ multiplied by ISI) [16, 17].

### CGM in all subjects

After OGTT, all subjects were monitored by CGMS (Medtronic MiniMed, Northridge, CA 91325, USA) for 72 h. The CGM system sensor was inserted in all subjects on day 0 and removed on day 3. Data were downloaded and glucose profiles were evaluated based on the data collected on days 1 and 2. The subjects were instructed to input at least four calibration readings per day and the times of key events. During the study, all subjects have standard meals provided by dietary division. The total calorie intake was 30 kcal/kg per day, with 50 % carbohydrates, 15 % proteins, and 35 % fats. The calorie distribution between breakfast, lunch, and dinner was 20, 40, and 40 %, respectively. Three daily meals were required to consume at time of 6:30–7:30, 11:30–12:30, and 18:00–19:00, respectively. All subjects were instructed to avoid strenuous exercise during CGM.

The parameters of glycemic variability included the standard deviation of blood glucose (SDBG), mean of daily continuous 24 h blood glucose (MBG), mean of daily differences (MODD), mean amplitude of glycemic excursions (MAGE) and the incremental areas above preprandial glucose values (AUC_pp_). MODD was calculated from the absolute difference between paired continuous glucose monitoring values during two successive 24 h periods and was used to assess inter-day glycemic variability [18]. MAGE, designed to quantify major swings of glycemia and to exclude minor ones, was used for assessing intra-day glycemic variability in this study [19]. AUC_pp_, calculated incremental areas of glucose above the each meal, was performed to evaluate the characteristics of postprandial glucose excursion [20]. It should be noted that MBG was a measure of overall of glycemic level and not specifically variability [21].

### Anthropometric indices and laboratory examination

Body mass index (BMI) was calculated (kg/m^2^). Systolic blood pressure (SBP) and diastolic blood pressure (DBP) taken three times using a sphygmomanometer and then averaged. Capillary glucose concentrations were measured with Lifescan Surestep blood glucose meter. Plasma glucose levels were measured using the glucose oxidase method. HbA1c was measured by high performance liquid chromatography (HPLC) with D-10 hemoglobin Testing Program (Bio-Rad). The serum insulin assay used magnetic beads-based enzymatic spectrofluorometric immunoassay with automatic enzyme immunoassay apparatus (AIA360, TOSOH). Serum glucose concentrations, total cholesterol (TC), triglyceride (TG), high density lipoprotein cholesterol (HDLC), and low density lipoprotein cholesterol (LDLC) were measured with Hitachi Model 7600 Series Automatic Analyzer.

### Statistical analyses

Data analyses were performed using the SPSS16.0 statistical software (SPSS Inc., USA). Continuous variables were expressed as mean ± standard deviation (SD) or median (interquartile range) in the case of skewed distributions. Categorical variables were described as frequency (percentage). The Student *t* test was applied to compare differences of continuous variables between the pGDM and control groups, nonparametric test (Mann–Whitney U test) was applied to compare non-normally distributed variables between the two groups, and Chi squared test was applied to compare categorical variables between the two groups. Relationship between glycemic variability and integrated *β* cell function was assessed using the Pearson’s correlation test and partial correlation test. *p* < 0.05 was considered to be statistically significant.

## Results

### Baseline characteristics in the subjects

As shown in Table 1, age, BMI and DBP were comparable between the pGDM and control groups (*p* > 0.05). SBP of pGDM group was higher than that in control group (*p* < 0.05). The prevalence of familial diabetes of pGDM group was higher than in control group (*p* < 0.05). TC, LDLC of pGDM group were higher than in control group (*p* < 0.05), HDLC of pGDM group was lower than in control group (*p* < 0.05), but there was no differences in TG between the two groups (*p* > 0.05). HbA1c of pGDM group was higher than that in control group (*p* < 0.05).

**Table 1:** Comparisons of clinical variables in pGDM and control groups

Variables	pGDM group	Control group	*t*	*p*
*n*	48	48	–	–
Age (year)	29.1 ± 4.1	29.8 ± 3.6	0.941	0.349
Familial diabetes, n (%)	7 (14.6)	0 (0.0)	–	0.012*
BMI (kg/m^2^)	26.8 ± 3.2	26.3 ± 2.9	0.768	0.444
SBP (mmHg)	130 ± 13	119 ± 15	3.502	0.001
DBP (mmHg)	76 ± 9	75 ± 10	0.493	0.623
TG (mmol/L)	1.5 (1.2–2.0)	1.3 (1.2–1.8)	–	0.442**
TC (mmol/L)	6.2 ± 1.0	4.8 ± 0.8	8.320	0.000
HDLC (mmol/L)	1.1 ± 0.3	1.3 ± 0.3	2.265	0.026
LDLC (mmol/L)	3.3 ± 0.9	2.4 ± 0.5	6.588	0.000
HbA1c (%)	5.8 ± 0.4	5.4 ± 0.3	8.617	0.000

Normally distributed values in the table are given as the mean ± SD, the non-normally distributed values are given as the median (25 and 75 % interquartiles)

pGDM group: NGT women with the previous GDM; Control group: NGT women without the previous GDM

*BMI* body mass index, *SBP/DBP* systolic/diastolic blood pressure, *TC* total cholesterol; *TG* triglyceride, *HDLC*: high density lipoprotein cholesterol, *LDLC* low density lipoprotein cholesterol, *HbA1c* glycosylated hemoglobin A1c

* Test with Fisher’s Exact test; ** Test with Mann–Whitney U test

### Changes of plasma glucose and insulin concentrations during the OGTT, and *β* cell functions derived from OGTT

The statistical comparisons of measurements characterizing the plasma glucose and insulin concentrations during the OGTT were summarized in Table 2. Plasma glucose levels at baseline, 30, 60 and 120 min after glucose ingestion were higher in pGDM group was higher than that in control group (*p* < 0.05), plasma glucose level at 180 min had no significantly difference between the two groups (*p* > 0.05). Insulin concentration at 60 min was significantly higher in pGDM group than in control group (*p* < 0.05), but there were no differences in insulin concentrations at baseline, 30, 120 and 180 min between the two groups (*p* > 0.05). The area under the glucose curve (AUC_glu_) was significantly higher in pGDM group than in control group (*p* < 0.05), but the area under the insulin curve (AUC_ins_) was comparable between the pGDM and control groups (*p* > 0.05).

**Table 2:** Comparisons of glucose and insulin concentrations during OGTT in pGDM and control groups

Variables	pGDM group	Control group	*t*	*p*
*n*	48	48	–	–
Glu0 (mmol/L)	5.5 ± 0.6	5.1 ± 0.5	3.845	0.000
Glu30 (mmol/L)	9.0 ± 1.7	7.5 ± 1.4	4.771	0.000
Glu60 (mmol/L)	8.8 ± 2.8	6.7 ± 2.3	4.071	0.000
Glu120 (mmol/L)	6.0 ± 1.1	5.4 ± 1.4	2.722	0.008
Glu180 (mmol/L)	4.9 ± 1.0	4.8 ± 1.0	0.677	0.500
Ins0 (miu/L)	6.3 (3.9–9.7)	5.6 (4.0–7.8)	–	0.391*
Ins30 (miu/L)	54.6 (36.4–88.7)	56.1 (37.9–77.0)	–	0.800*
Ins60 (miu/L)	63.1 (40.9–85.0)	44.1 (28.5–65.4)	–	0.011*
Ins120 (miu/L)	31.3 (14.9–42.6)	28.5 (21.6–40.1)	–	0.823*
Ins180 (miu/L)	9.2 (5.5–13.6)	8.1 (4.7–17.2)	–	0.496*
AUC_glu_ (mmol/L·h)	18.4 ± 2.8	15.7 ± 2.5	4.712	0.000
AUC_ins_ (miu/L·h)	114.7 (74.17–163.8)	99.6 (75.2–132.1)	–	0.356*
AUC_ins/glu_	5.6 (4.0–7.8)	6.6 (5.6–7.7)	–	0.031*
ISI	140.7 (81.9–190.0)	155.0 (120.3–205.7)	–	0.034*
ISSI-2	729.8 (496.9–943.0)	1027.2 (872.7–1177.5)	–	0.000*

Non-normally distributed values are given as the median (25 and 75 % interquartiles)

pGDM group: NGT women with the previous GDM; Control group: NGT women without the previous GDM

*Glut* plasma glucose concentrations at time *t* during OGTT, *Inst* plasma insulin concentrations at time *t* during OGTT, *AUC*_*glu*_ the area under the curve of glucose in 180 min, *AUC*_*ins*_ the area under the curve of insulin in 180 min, *ISI* insulin sensitivity index, *AUC*_*ins/glu*_ insulin secretion index, *ISSI-2* insulin secretion-sensitivity index-2

* Test with Mann–Whitney U test

After comparison of insulin sensitivity and insulin secretion index derived from OGTT, insulin sensitivity index (Matsuda ISI) and insulin secretion index (AUC_ins/glu_) in pGDM group were lower than in control group (*p* < 0.05) (Table 2). Integrated *β* cell function (ISSI-2) was significantly lower in pGDM group than in control group (*p* < 0.01).

### Glycemic variability in the subjects

**Fig. 1:**
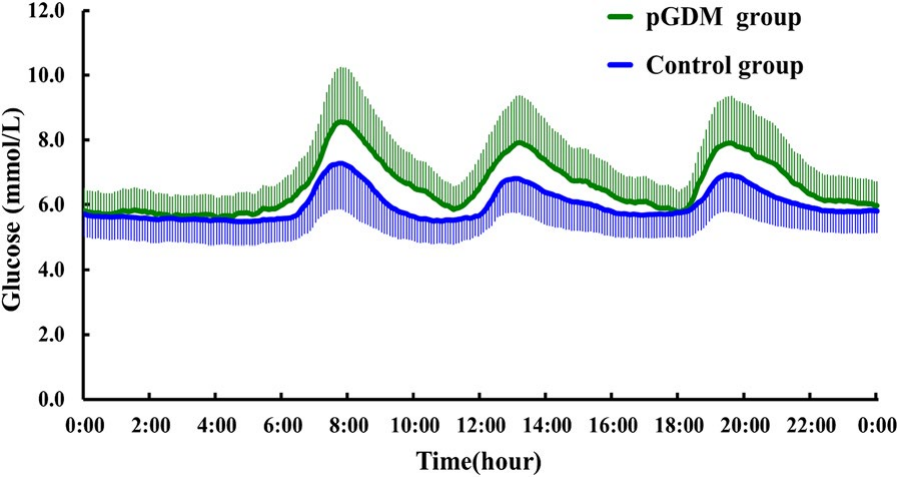
Continuous glucose profiles represented mean data from 24 h in pGDM and control groups

Glycemic variability detected from CGM system of pGDM and control groups were represented in Fig. 1. Although both pGDM and control groups were presented with NGT, the pGDM group had a greater MBG (6.5 ± 0.9 vs. 5.9 ± 0.8 mmol/L, *p* = 0.004), SDBG (1.3 ± 0.3 vs. 0.9 ± 0.2 mmol/L, *p* = 0.000), MODD (1.4 ± 0.3 vs. 1.1 ± 0.2 mmol/L, *p* = 0.002), MAGE (2.7 ± 0.4 vs. 1.8 ± 0.5 mmol/L, *p* = 0.000), and AUC_pp_ (26.8 ± 3.4 vs. 19.2 ± 3.2 mmol/L·h, *p* = 0.000), when compared to the control group (Table 3). And the differences remained significant after adjusting for age, familial diabetes, BMI, SBP, DBP, TG, TC, HDLC and LDLC.

**Table 3:** Comparisons of glycemic variability parameters in pGDM and control groups

Variables	pGDM group	Control group	*t*	*p*
*n*	48	48	–	–
MBG (mmol/L)	6.5 ± 0.9	5.9 ± 0.8	2.967	0.004
SDBG (mmol/L)	1.3 ± 0.3	0.9 ± 0.2	6.169	0.000
MODD (mmol/L)	1.4 ± 0.3	1.1 ± 0.2	3.186	0.002
MAGE (mmol/L)	2.7 ± 0.4	1.8 ± 0.5	8.696	0.000
AUC_pp_ (mmol/L·h)	26.8 ± 3.4	19.2 ± 3.2	11.267	0.000

Normally distributed values in the table are given as the mean ± SD

pGDM group: NGT women with the previous GDM; Control group: NGT women without the previous GDM

*MBG* mean of blood glucose, *SDBG* standard deviation of blood glucose, *MODD* mean of daily differences, *MAGE* mean amplitude of glycemic excursions, *AUC*_*pp*_ incremental areas above preprandial glucose values

### Relationships between glycemic variability and ISSI-2 in pGDM group

**Fig. 2:**
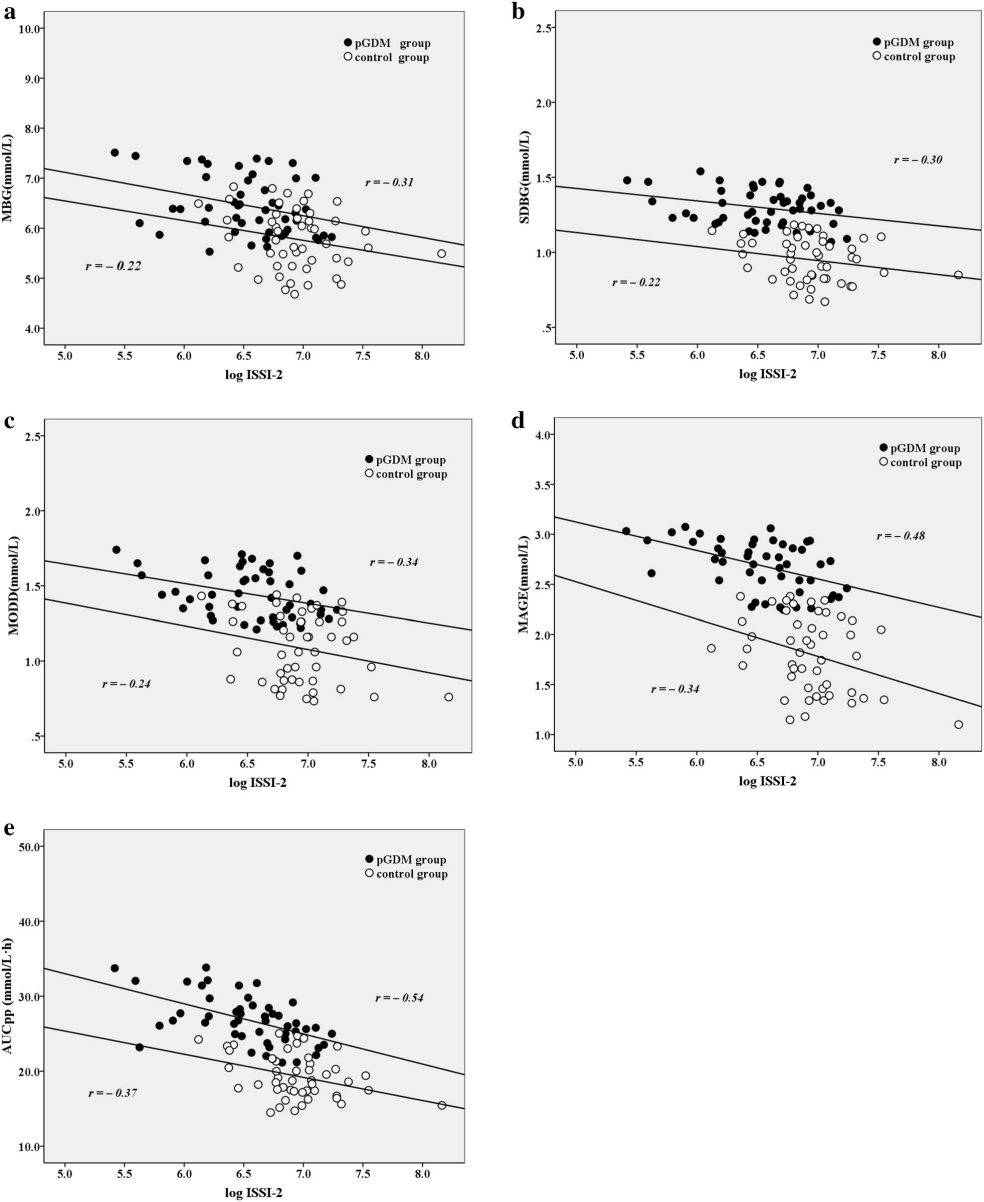
The relationships between glycemic variability parameters (**a** MBG, **b** SDBG, **c** MODD, **d** MAGE, **e** AUCpp) and ISSI-2 in pGDM and control groups

When the relationships between glycemic variability parameters and ISSI-2 were analyzed by Pearson’s correlation test, MBG (*r* = –0.31, *p* = 0.028), SDBG (*r* = −0.30, *p* = 0.037), MODD (*r* = −0.34, *p* = 0.017), MAGE (*r* = −0.48, *p* = 0.000) and AUC_pp_ (*r* = −0.54, *p* = 0.000) of pGDM group were all negatively associated with ISSI-2 in pGDM group (Fig. 2a–e). And the correlations remained significant after adjusting for age, familial diabetes, BMI, SBP, DBP, TG, TC, HDLC and LDLC.

## Discussion

In the specific group of pGDM women, our present study found that these women even with postpartum NGT were presented with elevated glycemic variability parameters. CGM can provide additional glycemic information compared to OGTT. Detailed 24 h glycemic profiles can be documented by CGM. Certain groups at high risks of diabetes, with so-called NGT status based on the OGTT, were presented with elevated glycemic variability. The study by Ma et al. [22] demonstrated that glycemic variability was increased in abdominally obese men with NGT. A study conducted in cystic fibrosis patients, it had been observed that normoglycemic subjects by OGTT had glucose excursions in the prediabetes as well as the diabetes range [9]. Madhu et al. [10] showed the normoglycemic obese first-degree relatives of type 2 diabetes had excursions into the higher dysglycemic range when studied by CGMS, and 90 % of subjects had excursions into the IGT range, and 15 % had excursions into the diabetes range. A1c-Derived Average Glucose (ADAG) study [23] showed 93 % of non-diabetic subjects exceeded the IGT threshold of 7.8 mmol/L, 7 % of them reached diabetes threshold of 11.1 mmol/L during CGMS, and the mean HbA1c in these subjects was within the normal range according to the ADA recommendations. Our previous study also showed that glycemic variability in normoglycemic subjects with elevated 1-h postload plasma glucose levels (NGT 1 h ≥ 8.6 mmol/L group) was higher than those in NGT 1 h < 8.6 mmol/L group [24]. Women with pGDM, and with postpartum NGT classified by the OGTT, had increased glycemic variability compared to NGT ones without pGDM in the present study. Glycemic variability parameters SDBG, MBG, MODD, MAGE and AUC_pp_ were elevated in women with pGDM. Our finding demonstrated the characteristics of glycemic variability in NGT women with pGDM.

The pGDM identified a population of young women predisposed for type 2 diabetes and related cardiovascular disease. Retnakaran et al. [25, 26] showed any degree of abnormal glucose homeostasis detected on antepartum screening for GDM should be associated with an increased risk of postpartum pre-diabetes, diabetes and latent metabolic syndrome. Elevated circulating markers of endothelial dysfunction in young women with a history of GDM could reflect an early stage on the pathway to the manifestation of future cardiometabolic disorders [27]. And Bo et al. [28] showed women with previous GDM have been shown to express early markers of vascular dysfunction such as increased intima-media thickness of carotid arteries(C-IMT). Zajdenverg et al. [29] showed microcirculation abnormality, evaluated by papillae rectification, was carried in young non-diabetic women with pGDM. Our study showed the normoglycemic women with pGDM were presented with elevated glycemic variability parameters, when compared with the women without pGDM. And elevated glycemic variability could be considered as a marker of metabolic abnormality of pGDM.

Glycemic variability could be an independent risk factor for vascular complications in addition to average glucose [30, 31]. Glucose variability parameters could be calculated with complex formulas designed specifically for the CGM data. In the previous studies, the SDBG around the mean glucose value was considered as a classical index to assess the glycemic variability [32]. MODD and MAGE are objective and valid indices to measure inter-day and intra-day glucose variability, respectively [18, 19]. AUCpp was performed to evaluate the characteristics of postprandial glucose excursion [20]. And several studies had demonstrated that glycemic variability, assessed by MAGE, was closely associated with micro- and macro-vascular complications [33, 34]. Our presented study demonstrated that glycemic variability parameters SDBG, MBG, MODD, MAGE and AUC_pp_ in pGDM group were higher than in the control group. Women with pGDM carried with early markers of vascular dysfunction [28, 29]. Hence, it implies that glycemic variability may be related to early markers of vascular dysfunction in women with pGDM. But it needs further study to document whether glycemic variability remission can improve the markers of vascular dysfunction in pGDM group.

GDM and type 2 diabetes shared a similar pathophysiology, characterized by deficiency in islet *β* cell secretion and insulin sensitivity, and GDM was a stress situation that may reveal predisposition to type 2 diabetes. Previous studies had shown that women with previous GDM even with postpartum normal oral glucose tolerance test had both insulin secretion and action defects [5, 6]. And our study showed insulin secretion and insulin sensitivity indices derived from OGTT were decreased in pGDM group than in control group. ISSI-2 proposed by Retnakaran et al. [16, 17] was a composite measure and may be a better index than either AUC_ins/glu_ or ISI alone to reflect the notion of declining *β* cell function and account for glycemic disorders. After correlation analyzing, SDBG, MBG, MODD, MAGE, and AUC_pp_ all negatively associated with the ISSI-2 in pGDM group. The decreased ISSI-2 of pGDM may be responsible for elevated glycemic variability.

It should be pointed out that our study had some limitations. First, the family history of diabetes could exaggerate the difference of glycemic variability between NGT women with and without previous GDM, but the comparison of glycemic variability between the two groups was adjusted for the familial diabetes. The pGDM group should be theoretically divided into subgroups with and without familial diabetes for further comparison, but the small sample size of subgroups might make some differences insignificant. Second, although we provided standard meals for subjects during the CGM system monitoring period, some factors, such as physical activity and emotional stress, etc., which may affect levels of glycemic variability, could not all be prevented. Third, we could not assess glycemic variability in relation to oxidative stress, inflammation and other markers of vascular dysfunction. The fourth limitation related to ISSI-2 was that circulating insulin levels during the OGTT may be affected by other factors apart from *β* cell function, such as incretin hormones and hepatic extraction. The two factors may limit the degree to which insulin levels during the OGTT can reflect *β* cell function.

## Conclusions

In summary, the glycemic variability parameters in NGT women with pGDM were higher than those in the NGT ones without pGDM, and elevated glycemic variability parameters may be associated with impaired *β* cell function.

## Abbreviations

NGT: normal glucose tolerance
Pgdm: previous gestational diabetes mellitus
BMI: body mass index
SBP/DBP: systolic/diastolic blood pressure
TC: total cholesterol
TG: triglyceride
HDLC: high density lipoprotein cholesterol
LDLC: low density lipoprotein cholesterol
HbA1c: glycosylated hemoglobin a1c
ISI: insulin sensitivity index
AUC_ins/glu_: insulin secretion index
ISSI-2: insulin secretion-sensitivity index-2
SDBG: standard deviation of blood glucose
MODD: mean of daily differences
MAGE: mean amplitude of glycemic excursions
AUC_pp_: incremental areas above preprandial glucose values
